# Cellular Angiofibroma of the Prostate: A Rare Tumor in an Unusual Location

**DOI:** 10.1155/2014/871530

**Published:** 2014-07-01

**Authors:** Inez Wyn, Maria Debiec-Rychter, Ben Van Cleynenbreugel, Raf Sciot

**Affiliations:** ^1^Department of Pathology, KU Leuven and University Hospitals Leuven, UZ Gasthuisberg, Herestraat 49, 3000 Leuven, Belgium; ^2^Human Genetics, KU Leuven and University Hospitals Leuven, 3000 Leuven, Belgium; ^3^Urology, KU Leuven and University Hospitals Leuven, 3000 Leuven, Belgium

## Abstract

We report the unusual occurrence of a cellular angiofibroma in prostatic tissue. In this case, a 84-year-old man presented in the emergency room with urinary retention. Ultrasound revealed an enlarged prostate, which was suggestive for benign prostatic hyperplasia. The patient was treated with a Millin retropubic prostatectomy. Macroscopically the prostate contained multiple circumscribed nodules. Microscopic examination of the tumor showed the appearance of cellular angiofibroma, consisting of bland spindle cells and prominent, hyalinized vessels. The diagnosis was supported by FISH, which revealed monoallelic loss of *RB1*/13q14 region, as seen in spindle cell lipoma, (extra-) mammary myofibroblastoma, and cellular angiofibroma. Cellular angiofibromas are rare, benign soft tissue tumours and were never reported in the prostatic gland.

## 1. Introduction

Cellular angiofibroma (CAF) is a rare benign mesenchymal lesion that was first described in 1997 by Nucci et al. [1] as a distinct mesenchymal neoplasm composed of 2 major components: spindle cells and prominent vessels. This original report presented four cases, all occurring in the vulvar region of middle aged woman. Since then, similar lesions have been described in the inguinoscrotal region of men, originally labeled as “angiomyofibroblastoma-like tumor of the male genital tract” [1, 2]. Different case reports reveal similar lesions in other sites: subcutaneous tissue of the chest wall [3], male pelvis [4], and oral mucosa [5], but an intraprostatic location was hitherto unreported.

## 2. Case Report

An 84-year-old man presented in the emergency room with urinary retention. Ultrasound revealed an enlarged prostate, suggestive for benign prostatic hyperplasia. The patient was treated with a Millin retropubic prostatectomy. Macroscopical examination showed prostatic tissue with firm to rubbery, white, circumscribed nodules.

**Figure 1:**
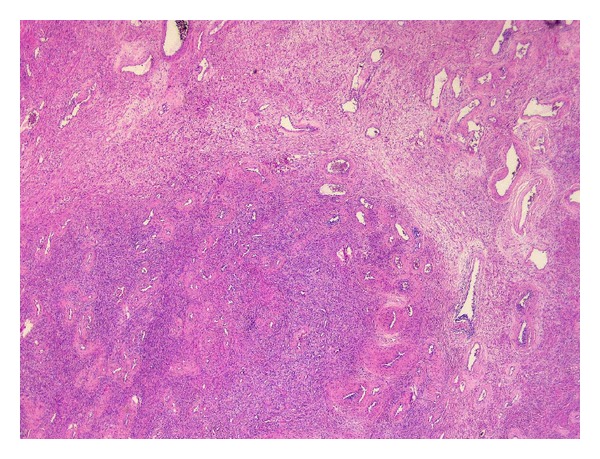
Low power view, showing the cellular spindle cell component and the hyaline vessels.

**Figure 2:**
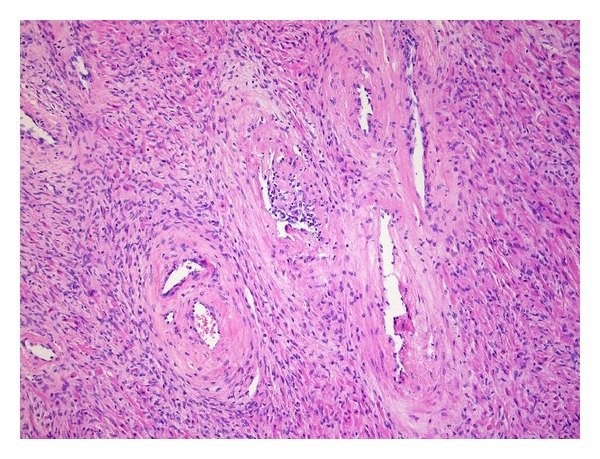
Detail of the blood vessels with hyalinized wall.

**Figure 3:**
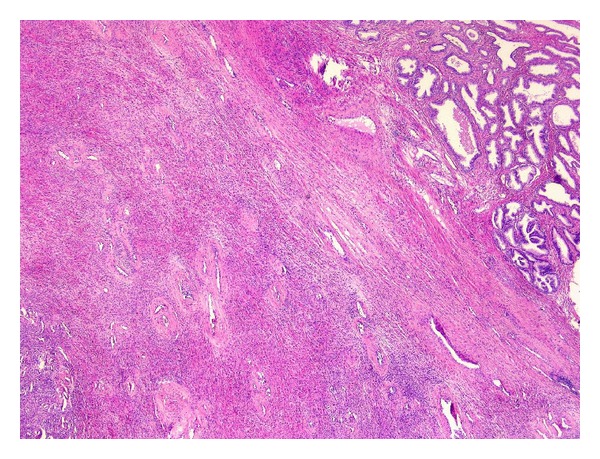
Prostatic tissue next to CAF.

Microscopic examination revealed a well-delineated nodular lesion with normal surrounding prostatic tissue (Figure 3). The lesion consisted of loosely arranged, bland spindle cells in a myxoid to collagenous stroma (Figure 1). Prominent vessels were present with marked hyalinization of the wall in some of them (Figure 2). Mitotic figures were absent. Morphologically, these features were suggestive for CAF.

**Figure 4:**
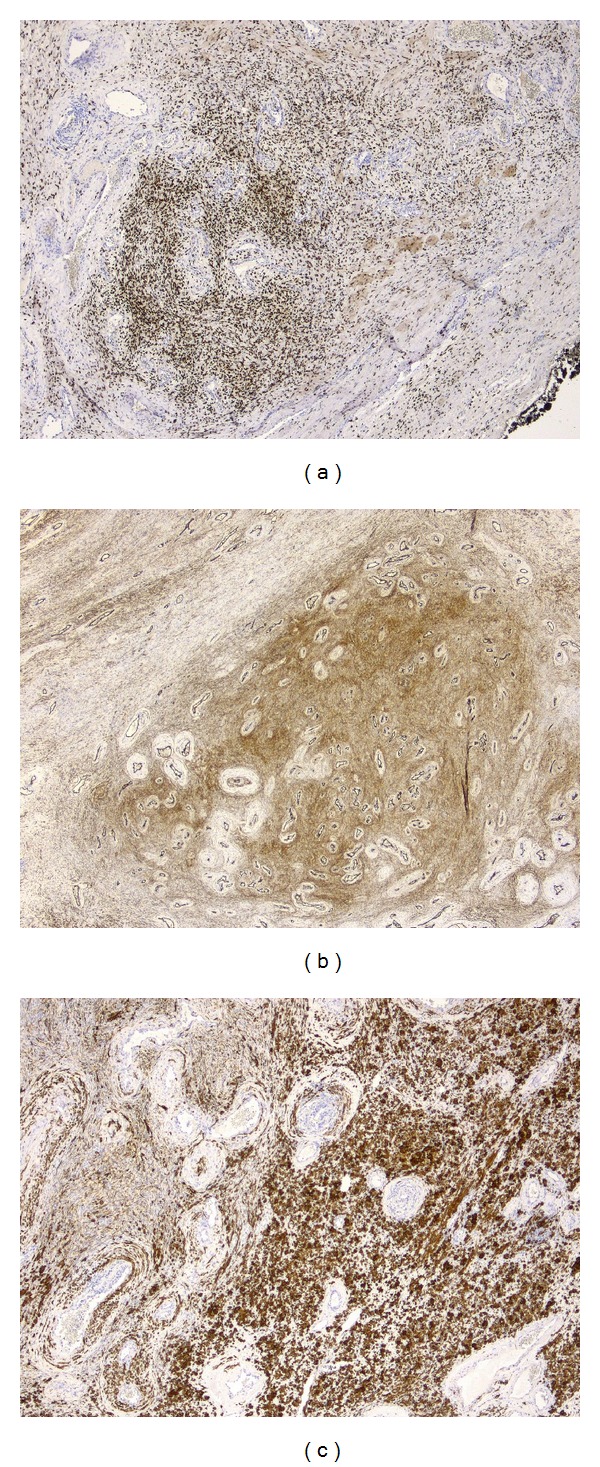
Positivity for PR (a), CD34 (b), and desmin (c).

Immunohistochemical analysis revealed strong positivity for alfa-sma, desmin, caldesmon, CD34, and progesteron receptor (PR) (Figure 4). CD31, ALK1, Myogenin, and estrogen receptor (ER) were negative.

Interphase dual-colour fluorescence in situ hybridization (FISH) was performed on 4 *μ*m formalin-fixed, paraffin embedded tumour sections, by a cohybridization of Spectrum Orange-labelled* RB1*/13q14 probe (LSI* RB1*-SO; Vysis Inc., Downers Grove, IL, USA) and a reference Spectrum Green-labelled chromosome X centromere probe (CEPX-SG, Vysis Inc.), as previously described [6]. The number of differentially labeled hybridization signals representing investigated gene and reference centromere X chromosomal regions were individually recorded for 100 nonoverlapped interphase nuclei, using a Zeiss microscope (Axioplan 2, Jena, Germany) equipped with the appropriate filters and CYTOVISION software. A cut-off of 30% was established for a positive result.

**Figure 5:**
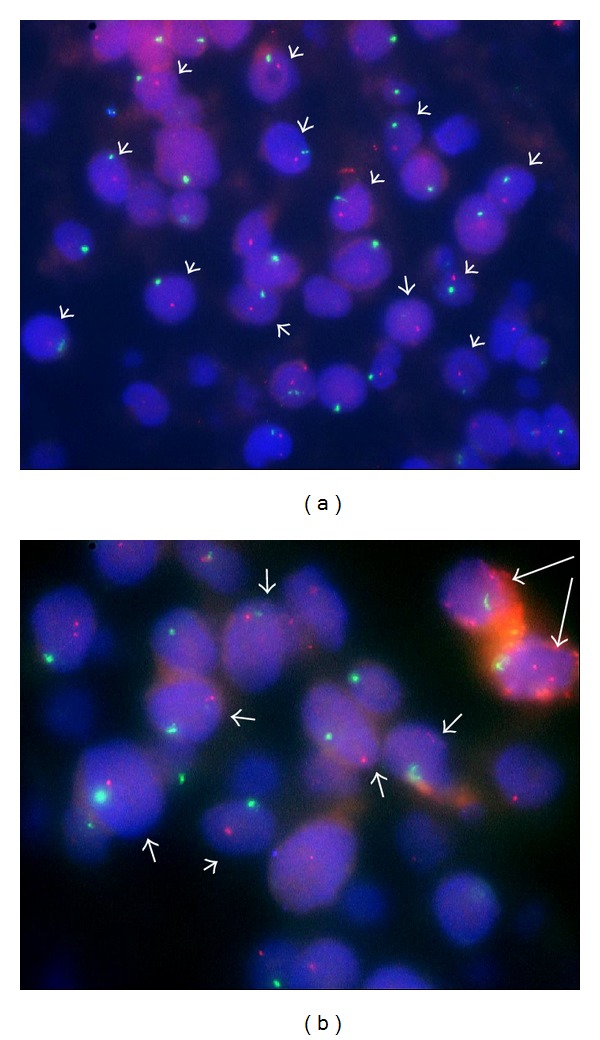
Double-colour FISH analysis of the tumour: loss of chromosome* RB1*/13q14 region as indicated by the presence of single red signals from Spectrum-Orange labelled* RB1* locus specific probe in reference to single green signals from Spectrum-Green labelled CEPX probe (short arrows). Representative tumour area under lower (a) and higher magnification (b). Long arrows on image (b) point to macrophages that show diploid* RB1* copy number, as expected in normal cells (internal control).

By FISH, 48% of nuclei demonstrated the monoallelic* RB1*/13q14 loci loss (Figure 5), as indicated by the presence of only one red signal in reference to chromosome X probe (green signal).

## 3. Discussion

Nonepithelial prostatic lesions are infrequent and cover a broad spectrum of benign and highly aggressive tumors.

CAFs are benign mesenchymal tumors, which occur equally in adult women and men [7]. Most often they occur in the vulvovaginal and inguinoscrotal region, but they are reported in other sites, including oral mucosa, male pelvis, and subcutaneous tissue of the chest wall [3–5]. In women they tend to occur earlier, most often in the fifth decade, while men are mostly affected in the seventh decade [7]. The tumor size in female patients is often smaller (median 2.8 cm) than that in male patients (median 7 cm) [7].

Ultrasound is often the initial imaging examination and reveals a well circumscribed, nodular lesion. To differentiate the lesion, MRI can be useful. MRI features of CAF are consistent with the pathological characteristics: a well circumscribed, benign cellular, and fibrous tumor with prominent vascularity [8].

Macroscopical examination of those lesions mostly reveal one or multiple nodules which are firm to rubbery in consistency and white, pale, or grey in color [9].

Immunohistological analysis reveals CD34 expression in approximately 66% of cases, together with alfa-sma and desmin positivity in a minority of tumors. They show positivity for ER and PR in 35–55%.

On morphology, inflammatory myofibroblastic tumor, solitary fibrous tumor, and cellular angiofibroma can mimic each other.

Solitary fibrous tumors (SFT) are mesenchymal lesions, which are also characterized by bland spindle cells, prominent blood vessels, with a typically branching “staghorn” pattern and hyalinization of the stroma. Like CAF, they also show CD34 positivity, but desmin expression is not a feature of SFT. Recently, nuclear STAT6 expression has been reported to be characteristic for SFT [10]. The differential diagnosis with stroma-predominant benign prostatic hyperplasia (BPH) and stromal tumor of uncertain malignant potential (STUMP) also has to be made.

STUMPS are tumors of specialized prostatic stroma in which definitive features of sarcoma are not identified. The tumor is often solitary and the proliferative stroma may infiltrate between benign glands. Mitotic activity is rare to absent. Necrosis is also absent. Four histological patterns are described: degenerative atypia, hypercellular stroma, myxoid, and phyllodes-type growth [11]. Only the last one shows a biphasic stromal and epithelial proliferation. The first three are purely mesenchymal proliferations. Cytological atypia is variable but more prominent in the first pattern. Immunohistochemically, STUMP expresses CD34 and muscle markers such as desmin and smooth muscle actin (SMA), like CAF do. They also can express progesteron receptor (PR). However, STUMP lacks the prominence of blood vessels, as described in CAF.

Benign prostatic hyperplasia typically has a multinodular growth and mostly arises from the transition zone (TZ). There is hyperplasia of both glandular and stromal tissues with papillary buds, infoldings, and cysts.

In benign prostatic hyperplasia abundant stromal capillaries are described with stromal cell condensation around the vessels. Fibromyxoid nodules can be seen within the lesion.

Inflammatory myofibroblastic tumor of the bladder is a well-recognized entity; however, prostatic inflammatory myofibroblastic tumor is extremely rare. Histologically, this tumor exhibits proliferation of uniform, bland spindle (myofibroblastic) cells of variable cellularity in a loose myxoid stroma. It often contains granulation tissue-type vascularity and a prominent inflammatory cell infiltrate. Immunohistochemically, these tumors coexpress alfa-sma, desmin, broad spectrum keratins, and low molecular weight keratins, but not CD34. ALK1 rearrangement and expression occurs in 20–75% of IMFs [11].

Genetic testing by FISH revealed a loss of RB1 (13q14-) in our tumor, which is described in spindle cell lipoma, (extra-) mammary myofibroblastoma, and cellular angiofibroma [7, 12, 13]. These entities also show morphologic overlap, but also subtle distinct features. The main features of cellular angiofibroma are the cellular spindle cell component and the presence of prominent, hyalinized blood vessels and minimal adipose tissue. The thick blood vessels are not seen in the other two entities. Spindle cell lipomas show a mixture of mature adipocytes and bland spindle cells in a mucinous, myxoid, or fibrous background. The spindle cells are arranged in short fascicles and associated with thick, ropy collagen bundles. In (extra-) mammary myofibroblastoma, fascicles of spindle cells having features of myofibroblasts are seen, with intervening hyalinized collagenous stroma and a variably prominent component of adipose tissue. This finding suggests that these tumors arise from a common stroma precursor cell, which undergoes (myo)fibroblastic or adipocyte differentiation [6].
